# Investigating the prevalence, predictors, and prognosis of suboptimal statin use early after a non-ST elevation acute coronary syndrome

**DOI:** 10.1016/j.jacl.2016.12.007

**Published:** 2017

**Authors:** Richard M. Turner, Peng Yin, Anita Hanson, Richard FitzGerald, Andrew P. Morris, Rod H. Stables, Andrea L. Jorgensen, Munir Pirmohamed

**Affiliations:** a Department of Molecular & Clinical Pharmacology, University of Liverpool, Liverpool, Merseyside, UK; b Department of Biostatistics, University of Liverpool, Liverpool, Merseyside, UK; c Liverpool Heart and Chest Hospital, Liverpool, Merseyside, UK

**Keywords:** Statin, Cardiovascular, Mortality, Discontinuation, Nonadherence, Muscular symptoms

## Abstract

**Background:**

High-potency statin therapy is recommended in the secondary prevention of cardiovascular disease but discontinuation, dose reduction, statin switching, and/or nonadherence occur in practice.

**Objectives:**

To determine the prevalence and predictors of deviation from high-potency statin use early after a non-ST elevation acute coronary syndrome (NSTE-ACS) and its association with subsequent major adverse cardiovascular events (MACE) and all-cause mortality (ACM).

**Methods:**

A total of 1005 patients from a UK-based prospective NSTE-ACS cohort study discharged on high-potency statin therapy (atorvastatin 80 mg, rosuvastatin 20 mg, or 40 mg daily) were included. At 1 month, patients were divided into constant high-potency statin users, and suboptimal users incorporating statin discontinuation, dose reduction, switching statin to a lower equivalent potency, and/or statin nonadherence. Follow-up was a median of 16 months.

**Results:**

There were 156 suboptimal (∼15.5%) and 849 constant statin users. Factors associated in multivariable analysis with suboptimal statin occurrence included female sex (odds ratio 1.75, 95% confidence interval [CI] 1.14–2.68) and muscular symptoms (odds ratio 4.28, 95% CI 1.30–14.08). Suboptimal statin use was associated with increased adjusted risks of time to MACE (hazard ratio 2.10, 95% CI 1.25–3.53, *P* = .005) and ACM (hazard ratio 2.46, 95% CI 1.38–4.39, *P* = .003). Subgroup analysis confirmed that the increased MACE/ACM risks were principally attributable to statin discontinuation or nonadherence.

**Conclusions:**

Conversion to suboptimal statin use is common early after NSTE-ACS and is partly related to muscular symptoms. Statin discontinuation or non-adherence carries an adverse prognosis. Interventions that preserve and enhance statin utilization could improve post NSTE-ACS outcomes.

## Highlights

• Deviation from high-potency statin therapy is common early in secondary prevention.
• Deviation can be by discontinuation, dose reduction, switching, or nonadherence.
• Muscular symptoms are associated with suboptimal statin use.
• Statin discontinuation/nonadherence is associated with increased adverse outcomes.
• Interventions to enhance statin use could improve secondary prevention outcomes.

## Introduction

Cardiovascular disease (CVD) is the leading cause of mortality worldwide.^1^, ^2^ In the United States and the United Kingdom, CVD accounts for the largest and second largest proportions of healthcare expenditure of any disease category, respectively.^3^, ^4^, ^5^ Although an acute coronary syndrome (ACS) is a sudden event, most of the morbidity and mortality accrues later, after hospital discharge. Statins are 3-hydroxy-3-methylglutaryl-Coenzyme A reductase inhibitors that reduce circulating low-density lipoprotein cholesterol. After an ACS, high-potency statin therapy, prescribed as atorvastatin 80 mg daily, is indicated because it has been demonstrated in randomized controlled trials (RCTs) to be highly effective and superior to both placebo and moderate statin therapy for reducing cardiovascular events.^6^, ^7^, ^8^ However, the effectiveness of drugs in RCTs can be undermined in clinical practice by several factors including poor adherence, discontinuation, and switching prescriptions to a lower equivalent potency. Poor statin adherence has been reported in up to 50% of patients,^9^ statin discontinuation rates vary from 15%^10^ to 60% to 75%^11^, ^12^ and changing to lower potency statin therapy has been noted in ∼1%^13^ to 42%^14^ of patients.

It is important to understand the clinical consequences of deviating from recommended high-potency statin therapy in high-risk patients who have had at least one cardiovascular event. The adverse effects of statin nonadherence and discontinuation on cardiovascular clinical outcomes have been investigated previously,^15^, ^16^, ^17^ but relatively little is known about the impact of statin dose reductions and/or switching to a statin of lower equivalent potency in real-world secondary prevention.^14^ The collective extent to which statin discontinuation, dose reduction, switching and/or nonadherence occur early in secondary prevention is also underreported. Furthermore, few real-world statin adherence studies have focused exclusively on non-ST elevation ACS (NSTE-ACS) patients, which as a group are often older, have more comorbidities, are more likely to receive noninterventional medical management, and have a worse long-term prognosis than patients suffering an ST-elevation myocardial infarction (MI)^18^, ^19^, ^20^ and so may be more susceptible to insufficient statin therapy.

Therefore, the aims of this study were to investigate (i) the prevalence of, (ii) the risk factors for, and (iii) the clinical consequences associated with conversion from high potency to “suboptimal” statin use due to statin discontinuation, dose reduction, switching to an alternative statin of lower equivalent potency and/or statin nonadherence, early after an NSTE-ACS in a contemporary prospective cardiovascular cohort.

## Materials and methods

### Prospective study outline

This investigation utilizes a prospective CVD observational study that was conducted at 16 different UK hospital sites between 2008 and 2013, entitled the Pharmacogenetics of Acute Coronary Syndrome (PhACS) study. A total of 1470 patients hospitalized with an NSTE-ACS (both non-ST elevation MI and unstable angina) were eligible for inclusion in PhACS. Patients were followed up at 1 (visit 2 [V2]) and 12 months (visit 3 [V3]) postrecruitment, and annually thereafter until all participants had been followed up for at least 12 months. Further study information is provided in the Supplement.

The protocol was approved by the Liverpool (adult) Research Ethics Committee, UK; site-specific approval was granted at all sites involved, and local informed consent was obtained from all study subjects in accordance with the Declaration of Helsinki.

### Cohort selection

Patients were eligible for inclusion in the present study if they were discharged on a high-potency statin from their index hospital NSTE-ACS admission. High-potency statin therapy was atorvastatin 80 mg daily, the equivalently potent rosuvastatin 20 mg, and rosuvastatin 40 mg daily (see eTable 1 in the Supplement for relative potency information). All other statins and doses were considered non–high-potency statin therapy. Patients were excluded if they died within 30 days of discharge because this prevented assessment of suboptimal statin status during follow-up (see below). Patients were excluded if their V2 occurred during a prolonged index hospital admission or did not actually occur until >180 days after index admission (as ∼85% of muscular symptoms occur within 180 days^21^), or they were lost to follow-up after V2.

### Assessment of statin adherence

At V2, cardiac medication adherence was assessed using the Brief Medication Questionnaire (BMQ; eFig. 1 in the Supplement).^22^ The BMQ incorporates three screens: a regimen screen, belief screen, and a recall screen. The BMQ has been compared with the Medication Events Monitoring System.^22^ The regimen screen had a sensitivity of 80% for detecting repetitive nonadherence and did not classify any adherent patients as nonadherent. However, it had 0% sensitivity for detecting sporadic nonadherence, and so its overall accuracy was 95%.^22^ Further information about the BMQ is available in the Supplement. For the main analysis, assessment of adherence utilized the regimen screen; patients were classed statin nonadherent if they reported missing at least 1 statin pill over the past week.

### Classification of suboptimal statin use

Patients were designated “suboptimal statin users” if, by V2, they had discontinued, reduced their statin dose, switched to an alternative statin of lower equivalent potency, and/or were statin nonadherent. Patients who were on high-potency statin therapy at baseline and V2 and were statin adherent represented “constant statin users.”

### Outcomes

(i) Suboptimal statin use at V2 was itself the outcome for investigating clinical factors associated with its occurrence.
(ii) For investigating potential sequelae of suboptimal statin use, the primary endpoint was time to first major adverse cardiovascular event (MACE): a composite of death from a CVD (or no known) cause or nonfatal MI or ischemic stroke. Time to all-cause mortality (ACM) was the secondary endpoint.

### Covariates

The following were considered for investigating factors associated with suboptimal statin occurrence: age ≥ 75 years, sex, body mass index ≥ 30, hypertension, hyperlipidemia, diabetes mellitus, smoking (current or previous vs nonsmokers), chronic kidney disease, chronic obstructive pulmonary disease, prior CVD (previous MI, stroke, transient ischemic attack or peripheral artery disease), statin use before index admission, raised index troponin, the high-potency statin on discharge (atorvastatin/rosuvastatin), treatment with percutaneous coronary intervention or coronary artery bypass graft surgery during or within 30 days after discharge from index admission, New York Heart Association functional class at V2, reported use at V2 of aspirin, a P2Y_12_ inhibitor, a beta blocker, an angiotensin-converting enzyme inhibitor or angiotensin II receptor blocker, warfarin, or a proton pump inhibitor (PPI), concomitant use of levothyroxine (a surrogate for hypothyroidism) or a drug(s) that inhibits cytochrome P450 3A4 (CYP3A4; listed in the Supplement), and muscular symptoms recorded at V2 (bothersome muscular pains/cramps/aches/weakness while on statin therapy recorded in the BMQ).

For the analyses investigating the risks of MACE and ACM, all the previously mentioned covariates were included except muscular symptoms, levothyroxine, CYP3A4-inhibiting drugs, and the type of high-potency statin discharged on. Follow-up commenced from the date of V2.

### Subgroup analyses

Suboptimal statin use was divided into those who had discontinued or were statin nonadherent and those who had reduced statin dose or switched statin (but were statin adherent), and the risks of time to MACE and ACM were analyzed for both subgroups, compared with constant statin users.

### Statistical analysis

(i) Imputation of missing data

Overall, 4.3% of data were missing, but 28.6% of cases had at least 1 missing value. This missing data were handled as follows. First, missing V2 dates were imputed by adding 30 days to baseline discharge date because 30 days represented the median duration of the nonmissing data. Second, V2 drug data were manually imputed where possible by comparison of baseline and V3 drug data. Missing V2 muscular symptoms were also manually imputed as “no symptoms” because only 1.2% of patients openly reported symptoms. Finally, multiple imputations were used: all remaining missing values were sampled using a fully conditional specification method, which uses an iterative Markov chain Monte Carlo procedure, and 10 imputation datasets were generated. See the Supplement for further details.

(ii) Investigating factors associated with suboptimal statin use.

After imputation, the null hypothesis of no association with suboptimal statin occurrence (compared with constant statin use) was tested for each variable using the Wald test because it generates a pooled value from the 10 datasets. Those covariates with univariate *P* < .1 were entered into a multivariable logistic regression model, using forward stepwise (likelihood ratio) selection. Odds ratios (ORs) and *P* values are pooled from the 10 imputed datasets; *P* < .05 indicated significance.

(iii) Investigating risks of MACE and ACM associated with suboptimal statin use

A univariate Cox proportional hazard model was fitted for each covariate to test its association with time to MACE; the same was performed for time to ACM. For each covariate, the Cox proportional hazards assumption was assessed by visual inspection of Kaplan–Meier curves. If a covariate did not meet the proportional hazards assumption, it was excluded from the main analyses (see sensitivity analyses D1 and D2). Covariates meeting the proportional hazards assumption and *P* value < .1 in univariate analysis were taken forward into multivariable Cox proportional hazards modeling, with the final multivariable model covariates chosen by forward stepwise (likelihood ratio) selection. After the covariate model had been fitted for both time to MACE and time to ACM, suboptimal statin use was introduced into both models to test its adjusted association with risk of MACE or ACM. The hazard ratios (HRs) and *P* values provided in the results section are pooled results across all imputed datasets, except in the complete cases sensitivity analyses.

As two outcomes (MACE and ACM) were investigated here, a Bonferroni correction was used to adjust the significance threshold to *P* ≤ .025. This threshold was also applied to all sensitivity analyses that further examined the risks of MACE or ACM associated with suboptimal statin use (see the following).

### Sensitivity analyses

To investigate result robustness, sensitivity analyses were undertaken (see the Supplement for further details). First, a subcohort consisting of all cases with complete data (“complete cases”) assessed whether missing data impacted either the factors associated with suboptimal statin occurrence or the associations between suboptimal statin use and risk of MACE/ACM. Additional sensitivity analyses evaluated the robustness of the associations between suboptimal statin use and MACE/ACM further by expanding the statin nonadherence definition, considering covariates that did not meet the proportional hazards assumption for full follow-up duration, including all variables that differed significantly between suboptimal and constant statin user groups at V2, and examining the potential for healthy user bias by considering PPI prescription changes between baseline discharge and V2.

The expanded statin nonadherence definition was: patients that missed at least 1 statin pill (BMQ Qu. 1e), took a statin for 6 or fewer days (Qu. 1b; both from regimen screen), reported that the statin did not work well for them or they did not know (Qu. 1g), found that the statin bothered them at least a little (Qu. 2; both from belief screen), and those that found it at least somewhat hard to remember to take all their pills (Qu. 3c; the recall screen).

All analyses were performed using IBM SPSS version 22.0 (IBM Corp, Armonk, NY, USA).

## Results

**Figure 1:**
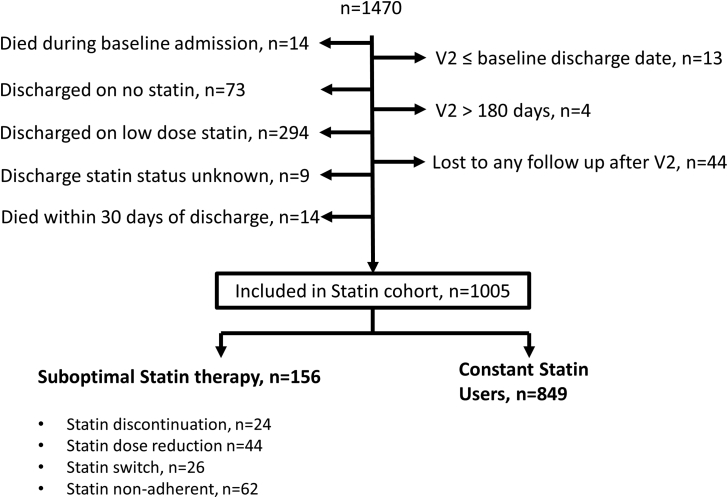
A schematic of the study selection process.

Figure 1 outlines the cohort selection process for this study. A total of 1005 patients discharged on a high-potency statin were included; >99% were prescribed atorvastatin 80 mg daily. One hundred fifty-six patients (15.5%) were suboptimal statin users by V2; 849 (84.5%) remained on and adherent to high-potency statin therapy, constituting constant statin users.

Of 1005 eligible patients discharged from hospital with a diagnosis of non-ST elevation ACS on recommended high-potency statin therapy, 156 (15.5%) had inadequate statin utilization by a median of 1 month after hospital discharge; 849 (84.5%) patients remained on and adherent to high-potency statin therapy at V2.

### Factors associated with suboptimal statin occurrence

Suboptimal and constant statin users were broadly similar (Table 1). However, in multivariable logistic regression, being female (*P* = .010), not on either a P2Y_12_ inhibitor (*P* = .007) or beta blocker at V2 (*P* = .036), and being bothered by muscular symptoms (*P* = .017) were all associated with an increased adjusted risk of suboptimal statin occurrence (Table 2).

### Risks of MACE and ACM associated with suboptimal statin use

**Figure 2:**
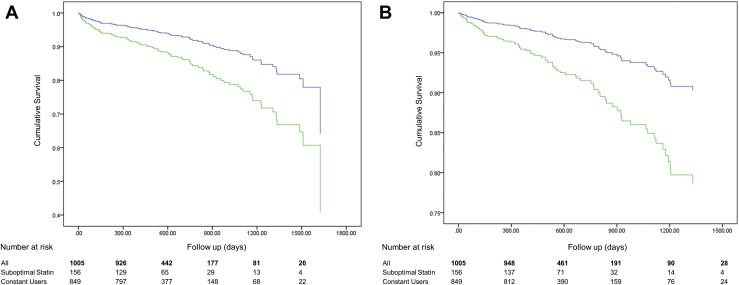
Cumulative survival curves. The cumulative survival curves compared suboptimal statin (green) and constant statin use (blue) group survival free from; (A) major adverse cardiovascular events (MACE) and (B) all-cause mortality (ACM). Survival curves plotted until last event occurrence. (Color version of figure is available online.)

The median study duration after V2 was 16 months, and there were 113 MACE and 79 ACM events; 33% of ACM deaths were noncardiovascular. Table 3 shows the results of the univariate analyses of association between time to MACE, or time to ACM, and each variable considered. Of patients with suboptimal statin use, 32 and 25 suffered MACE and ACM, respectively. In multivariable analysis, suboptimal statin use was a risk for both time to MACE (HR 2.10, 95% confidence interval [CI] 1.25–3.53, *P* = .005) and time to ACM (HR 2.46, 95% CI 1.38–4.39, *P* = .003), after adjusting for age ≥ 75 years, prior CVD, percutaneous coronary intervention or coronary artery bypass graft surgery treatment, New York Heart Association class, and either diabetes mellitus (time to MACE) or chronic kidney disease (time to ACM; Table 4). The adjusted survival curves, stratified by suboptimal statin status, are illustrated in Figure 2A and B, and demonstrate early separation of hazard risk after V2.

### Sub-group analyses

The subgroup of suboptimal statin users that had discontinued/were nonadherent (*n* = 95) had significantly increased risks of MACE (HR 2.74 [CI 1.49–5.04], *P* = .001) and ACM (HR 3.50 [CI 1.69–7.23], *P* = .001), compared with constant statin users (Table 5). The smaller subgroup of adherent patients with reduced statin dose/switched statin (*n* = 61) did not have significantly increased risks of MACE (*P* = .24) or ACM (*P* = .22; Table 5).

### Sensitivity analyses

Complete cases subcohort sensitivity analyses reinforce that muscular symptoms, female sex, and beta blocker use were associated with suboptimal statin occurrence (eTables 2, 3 in the Supplement). Suboptimal statin use was robustly associated with risks of MACE, and ACM, irrespective of adherence definition (Table 5), missing data imputation (Table 5, and eTable 5 in the Supplement), variables that did not meet the proportional hazards assumption (P2Y_12_ use for MACE and sex for ACM) and after inclusion of all variables associated with suboptimal statin occurrence (eTables 5–9 in the Supplement). There was no substantive healthy user effect (eTables 5, 10, 11 in the Supplement).

## Discussion

The main findings of this study are first, by a median of 1 month after admission for NSTE-ACS in patients discharged on high-potency statin therapy, ∼15% have suboptimal statin utilization. Expanding the nonadherence definition increased this to 27% (Table 5). Second, suboptimal statin occurrence was associated with muscular symptoms, female sex, and reduced use of beta blockers and P2Y_12_ inhibitors. Third, suboptimal statin use was associated with increased adjusted risks of times to both MACE and ACM, although this was largely attributable to statin discontinuation/nonadherence early after NSTE-ACS rather than statin dose reduction/statin switching.

This study is novel because it considered all components of attenuated statin therapy (discontinuation, nonadherence, switching, and dose reduction), both collectively and in subgroups. To date, most adherence studies have assessed medication availability (eg, proportion of days covered) via electronic data sources.^23^, ^24^, ^25^ Although this approach allows assessment of average adherence over time, it is difficult for healthcare professionals to easily measure and act on in practice. Importantly, the pragmatic approach used in this study highlights the importance of assessing statin usage early after hospital discharge in CVD secondary prevention patients. Furthermore, the assessment of statin utilization used in this study is relatively straightforward and so is potentially actionable.

Overall, there were few differences at V2 between suboptimal and constant statin users. However interestingly, females^23^, ^24^, ^26^, ^27^ and a lower rate of beta blocker^23^, ^28^ and antiplatelet^29^ drug use have all previously been associated with poorer statin adherence. In this study, suboptimal statin users were more likely to have not been prescribed P2Y_12_ therapy at hospital discharge and to have stopped the beta blocker they were discharged on (data not shown). This study also found that muscular symptoms were a risk factor for suboptimal statin use. Very few other statin utilization studies have included potential adverse events, although a cross-sectional Internet-based survey previously determined that muscular symptoms are reported more frequently in patients that have discontinued, switched statin, or are nonadherent, compared with nonswitching statin adherent participants.^27^ Overall, there was no evidence that these differences altered the increased risks of time to MACE or ACM associated with suboptimal statin use (eTables 8 and 9 in the Supplement).

Statins are associated with increased myotoxicity, incident diabetes mellitus and probably hemorrhagic stroke.^30^ Statin-associated muscular symptoms are reported in ∼1.5% to 3% of statin users in RCTs^31^ and in ∼7%–29% of patients in observational studies.^32^ However, while rare statin-induced severe myopathy/rhabdomyolysis is incontrovertible, the contribution of statins to milder muscle symptoms remains controversial. One informative estimate for the extent of muscular symptoms attributable to statin therapy is ∼5%,^33^ which is derived from a blinded RCT that compared rates of stringently defined myalgia in healthy volunteers receiving either atorvastatin 80 mg daily or placebo for 6 months (*P* = .05).^33^ The reported rate of bothersome muscular symptoms in our observational study was low (∼1.2%; Table 1). This may be a reflection of muscular symptoms not being explicitly asked about and/or because patients who experienced muscular symptoms shortly after discharge had amended their statin therapy by V2, with potential symptomatic resolution. There is currently no unifying mechanistic explanation for statin-induced myotoxicity. However, several factors increase risk including female sex, advanced age, hypothyroidism, chronic kidney disease, exercise, drug–drug interactions, and for simvastatin myopathy specifically a genetic variant (*SCLO1B1* rs4149056) is a risk factor.^34^

The largest type of suboptimal statin users in this study was statin nonadherent patients. The etiology of statin nonadherence is multifactorial and incompletely understood; predictors beyond those identified in this study include age, low income, and increased noncardiovascular medications.^35^ Health beliefs and knowledge affect both perceptions of need for a treatment, and counteracting perceptions of potential treatment adverse effects, are influenced by factors such as patient satisfaction with physician treatment explanations, and likely also modulate nonadherence.^36^ Therefore, irrespective of the exact underlying etiology of mild muscular symptoms, the attribution of these symptoms to statin therapy by a patient will potentially reduce statin utilization.

Another potential reason for the statin discontinuation/dose reductions/statin switching observed in this study early after an NSTE-ACS is a communication breakdown leading to the high-potency statin hospital discharge prescription not being transferred and incorporated into a patient's repeat outpatient prescription drug list. Transfer of medical information from secondary to primary care is often incomplete and untimely,^37^, ^38^ although further research is required to evaluate the extent of its potential impact on early post-ACS suboptimal statin therapy.

Previous secondary prevention cohorts have reported elevated risk estimates for statin nonadherence or discontinuation/persistence of 1.01–5.26 for MACE and 1.25–5.00 for mortality, with the majority reporting statistically significant results.^39^ Our study results of increased adjusted risks of time to MACE or ACM associated with both suboptimal statin use and the statin nonadherence/discontinuation subgroup in particular are in keeping with these findings. This emphazises the generalizability of these clinically relevant findings across secondary prevention populations, settings, and study designs.

In this study of NSTE-ACS patients, the statin dose reduction/switching statin subgroup was not significantly associated with increased risks of time to MACE or ACM. One other prospective study has investigated statin dose reduction/switching after ACS, but included both NSTE-ACS and ST-elevation ACS patients and reported a significantly increased risk for adverse clinical outcomes (HR 2.7, 95% CI 1.7–5.1).^14^ Our smaller number of dose reduction/switching cases (*n* = 61) may have accounted for this subgroup only showing a nonsignificant trend for increased risk. Two other observational studies have investigated the influence of switching from atorvastatin to simvastatin^40^, ^41^ on cardiovascular events, using mixed primary/secondary prevention populations identified using electronic healthcare databases. The UK-based study found a modestly increased cardiovascular event risk (HR 1.30, 95% CI 1.02–1.64),^40^ whereas the US-based study found no association.^41^ However, in both these studies, most patients were on atorvastatin ≤20 mg/day, and it has been noted that the proportion of switches from atorvastatin to a lower rather than equivalently potent simvastatin regimen increases as the initial atorvastatin dose increases.^41^ This is particularly relevant in post-ACS patients, as practically all switches from atorvastatin 80 mg/day are to another statin of lower equivalent potency. Overall, persistent adherence to high-potency statin therapy after an ACS appears optimal; however, if necessary, reducing the dose or switching statin appears preferable to statin nonadherence or complete discontinuation.

Recently, several interventions have been proposed that attempt to reduce nonadherence/discontinuation and improve statin therapeutic effectiveness, including improving CVD and statin literacy, co-payment reduction, using fixed-dose “polypill” combinations and behavior-modification interventions.^17^ For example, brief pharmacist-led face-to-face counseling sessions have been shown to improve statin adherence.^42^ There is also increasing interest in utilizing mobile technology applications (apps) to remind patients to take their medications, and patients are being involved in medication-related app development.^43^ It is thus plausible that an intervention based on reminders (eg, apps and/or posted letters) and face-to-face contact could be targeted to patients early after a CVD event to both screen for and address suboptimal statin utilization, although further research is required.

Our study has limitations. It is a *post hoc* assessment of the PhACS study. The exact reasons for statin prescription changes and the cause(s) for patient nonadherence were not recorded. The data are observational and, therefore, we cannot confirm causality because of the potential for confounding influences by unmeasured variables, such as cardiac rehabilitation attendance. Although we cannot definitively exclude any healthy user effect, our assessment of PPI utilization (eTables 10 and 11 in the Supplement) is in keeping with the lack of healthy user effect reported in other statin utilization studies^23^, ^24^, ^44^ and so makes a prominent contribution of this type of influence unlikely. It is acknowledged that both the assessment of statin adherence at a single time point and basing the primary assessment on the number of pills missed over the preceding week will limit detection of sporadic nonadherence.^22^ However, the expanded nonadherence definition (Table 5) includes all components of the BMQ and the BMQ recall screen (enquiring about how hard the patient finds it to remember to take all the pills) has a sensitivity of 90% for sporadic nonadherence, albeit with a reduced specificity of 80%.^22^ The assessment of statin utilization at 1 month is also unlikely long enough to capture full stabilization of drug use. However, median statin discontinuation in secondary prevention appears to occur at 30 to 37 days after discharge,^14^, ^45^ and our approach does not preclude follow-up adherence assessments. Overall, this investigation used a prospective multicenter study with event validation rather than electronic diagnostic codes, and the several sensitivity analyses confer robustness to the main findings.

In conclusion, patients with an NSTE-ACS are at high risk of subsequent MACE and ACM. After discharge on high-potency statin therapy, the intensity of statin therapy is already reduced for a sizeable proportion of patients by 1 month back in the community, and self-reported muscular symptoms appear to increase the risk for suboptimal statin utilization. Early statin discontinuation/nonadherence correlates with increased risks of subsequent MACE and ACM. Physicians, pharmacists, and cardiac rehabilitation programs are encouraged to discuss statin therapy with ACS patients early after discharge, reaffirm the benefits of statins, and explore barriers to their effective use to maintain and enhance statin utilization and so potentially improve post NSTE-ACS outcomes.

## Figures and Tables

**Table 1:** Characteristics of suboptimal and constant statin users

Variable	Suboptimal statin therapy	Constant statin users	Unadjusted *P* value
Patients (%)	156 (15.6)	849 (84.4)	
Median follow-up from V2 (mo)	16	15	.52
Demographics			
Age ≥ 75 y, *n* (%)	39 (25.0)	161 (19.0)	.13
Men, *n* (%)	102 (65.4)	660 (77.4)	.004
BMI ≥ 30, *n* (%)	54 (34.6)	292 (33.4)	.92
Medical history, *n* (%)			
Hypertension	93 (59.6)	490 (57.7)	.63
Hyperlipidemia	75 (48.1)	455 (53.6)	.27
Diabetes mellitus	43 (27.6)	43 (27.6)	.091
Ever smoked	113 (72.4)	588 (69.3)	.42
CKD (Cr > 150 μmol/L)	13 (8.3)	48 (5.7)	.28
COPD	13 (8.3)	74 (8.7)	.89
Prior CVD∗	51 (32.7)	287 (33.8)	.82
On statin before index admission	79 (50.6)	387 (45.6)	.30
Diagnosis, *n* (%)†			
Troponin-raised NSTE-ACS	149 (95.5)	828 (97.5)	.16
Normal troponin NSTE-ACS	7 (4.5)	21 (2.5)	–
Treatment, *n* (%)			
PCI/CABG	72 (46.2)	401 (47.2)	.80
Discharged on atorvastatin 80 mg daily	155 (99.4)	843 (99.3)	.91
NYHA functional classification at Visit 2, *n* (%)			
Class I	82 (52.6)	457 (53.8)	.61
Class II	56 (35.9)	314 (37.0)	
Class III	18 (11.5)	70 (8.3)	
Class IV	0 (0.0)	8 (0.9)	
Drugs at Visit 2, *n* (%)			
Aspirin	142 (91.0)	795 (93.6)	.36
P2Y_12_ inhibitor	122 (78.2)	738 (86.9)	.006
Beta blocker	119 (76.3)	725 (85.4)	.016
ACEI/ARB	121 (77.6)	706 (83.2)	.11
Warfarin	6 (3.9)	41 (4.8)	.57
Proton pump inhibitor	67 (43.0)	358 (42.2)	.89
CYP3A4-inhibitors	19 (12.2)	66 (7.8)	.080
Levothyroxine	6 (3.8)	39 (4.6)	.67
Muscular symptoms at V2, *n* (%)	5 (3.2)	7 (0.8)	.020

ACEI, angiotensin-converting enzyme inhibitor; ARA, aldosterone receptor antagonist; ARB, angiotensin II receptor blocker; BMI, body mass index; CABG, coronary artery bypass graft surgery; CKD, chronic kidney disease; COPD, chronic obstructive pulmonary disease; Cr, creatinine; CVD, cardiovascular disease; CYP3A4, cytochrome P450 3A4 drug-metabolizing enzyme; LD, loop diuretic; NSTE-ACS, non-ST elevation acute coronary syndrome; NYHA, New York Heart Association; PCI, percutaneous coronary intervention; V2, Visit 2.

∗ Prior CVD encompasses past myocardial infarction, stroke, transient ischemic attack or peripheral artery disease.

† Raised troponin taken to indicate non-ST elevation myocardial infarction and a normal troponin unstable angina.

**Table 2:** Adjusted factors associated with suboptimal statin occurrence

Risk factor	Suboptimal statin therapy, *n* (%)	Constant statin users, *n* (%)	Multivariable analysis	
			OR (95% CI)	*P* value
Muscular symptoms	5 (3.2)	7 (0.8)	4.28 (1.30–14.08)	.017
Sex (female vs male)	M: 102 (65.4)	M: 660 (77.4)	1.75 (1.14–2.68)	.010
P2Y_12_ inhibitor at V2	122 (78.2)	738 (86.9)	0.53 (0.34–0.84)	.007
Beta blocker at V2	119 (76.3)	725 (85.4)	0.59 (0.36–0.96)	.036

CI, confidence interval; OR, odds ratio.

Covariates with univariate *P* < .1 were entered into multivariable logistic regression modeling using a forward likelihood ratio method to select the multivariable model presented here.

**Table 3:** Univariate Cox regression analysis results for association with time to MACE or time to ACM

Variable	Time to MACE (*n* = 113)		Time to ACM (*n* = 79)	
	HR (95% CI)	*P* value	HR (95% CI)	*P* value
Demographics				
Age ≥ 75 y	3.02 (2.07–4.40)	<.001	5.17 (3.31–8.07)	<.001
Sex (female vs male)	1.31 (0.87–1.97)	NS (*P* = .19)	∗	∗
BMI ≥ 30	1.30 (0.89–1.90)	NS (*P* = .18)	1.40 (0.89–2.20)	NS (*P* = .14)
Medical history				
Hypertension	1.82 (1.21–2.71)	.004	2.12 (1.29–3.49)	.003
Hyperlipidemia	1.56 (1.06–2.27)	.023	1.90 (1.20–3.02)	.007
Diabetes mellitus	2.56 (1.76–3.74)	<.001	2.77 (1.78–4.33)	<.001
Ever smoked	1.22 (0.80–1.86)	NS (*P* = .35)	1.33 (0.80–2.21)	NS (*P* = .27)
CKD (Cr > 150)	2.72 (1.65–4.47)	<.001	3.93 (2.34–6.61)	<.001
COPD	1.39 (0.79–2.43)	.26	1.88 (1.03–3.42)	.039
Prior CVD	3.06 (2.09–4.48)	<.001	4.25 (2.64–6.87)	<.001
On statin before index admission	1.66 (1.14–2.42)	.009	2.01 (1.26–3.21)	.003
Diagnosis				
Raised vs normal troponin NSTE-ACS	0.84 (0.34–2.09)	NS (*P* = .71)	1.47 (0.36–6.01)	NS (*P* = .59)
Treatment				
PCI/CABG	0.42 (0.28–0.63)	<.001	0.31 (0.18–0.53)	<.001
Functional statin at V2				
NYHA	1.89 (1.51–2.37)	<.001	2.07 (1.60–2.70)	<.001
Drugs at V2				
Suboptimal statin therapy	2.18 (1.40–3.40)	.001	2.54 (1.56–4.14)	<.001
Aspirin	0.49 (0.28–0.86)	.013	0.23 (0.13–0.38)	<.001
P2Y_12_ inhibitor	∗	∗	0.66 (0.39–1.12)	NS (*P* = .12)
Beta blocker	0.86 (0.53–1.42)	NS (*P* = .56)	0.76 (0.43–1.34)	NS (*P* = .34)
ACEI/ARB	1.46 (0.84–2.55)	NS (*P* = .18)	1.16 (0.63–2.13)	NS (*P* = .63)
Warfarin	2.23 (1.13–4.42)	.022	2.94 (1.41–6.13)	.004
Proton pump inhibitor	0.97 (0.67–1.42)	NS (*P* = .89)	1.40 (0.90–2.18)	NS (*P* = .14)

ACEI, angiotensin-converting enzyme inhibitor; ACM, all-cause mortality; ARB, angiotensin II receptor blocker; BMI, body mass index; CABG, coronary artery bypass graft surgery; CI, confidence interval; CKD, chronic kidney disease; COPD, chronic obstructive pulmonary disease; CVD, cardiovascular disease; HR, hazard ratio; MACE, major adverse cardiovascular events; NSTE-ACS, non-ST elevation acute coronary syndrome; NYHA, New York Heart Association; PCI, percutaneous coronary intervention.

∗ Visit 2 P2Y_12_ status did not meet the proportional hazards assumption for MACE, and patient sex did not meet the proportional hazards assumption for ACM; these variables were considered in sensitivity analyses (see eTables 5, 8, 9 in the Supplement).

**Table 4:** Multivariable-adjusted Cox regression results for risk of time to MACE or ACM

Variable	Time to MACE		Time to ACM	
	HR (95% CI)	*P* value	HR (95% CI)	*P* value
Suboptimal statin therapy	2.10 (1.25–3.53)	.005	2.46 (1.38–4.39)	.003
Age ≥ 75 y	2.05 (1.36–3.09)	.001	3.47 (2.12–5.68)	<.001
NYHA	1.48 (1.12–1.96)	.006	1.62 (1.16–2.27)	.005
Treatment with PCI/CABG	0.56 (0.37–0.86)	.008	0.49 (0.28–0.85)	.011
Prior CVD	2.00 (1.31–3.04)	.001	2.43 (1.45–4.08)	.001
Diabetes mellitus	1.52 (1.002–2.30)	.049	–	–
Chronic kidney disease	–	–	1.65 (0.93–2.93)	.089

ACM, all-cause mortality; CABG, coronary artery bypass graft surgery; CI, confidence interval; CVD, cardiovascular disease; HR, hazard ratio; MACE, major adverse cardiovascular events; NYHA, New York Heart Association; PCI, percutaneous coronary intervention.

Covariates with *P* < .1 in univariate Cox analysis were entered into multivariable Cox regression modeling using the forward likelihood ratio method to select the covariate model (variables not in bold font). After these times to MACE or ACM covariate models were selected, the suboptimal statin therapy variable was entered into both models to produce the presented results.

**Table 5:** Summary of main results for the adjusted risks of time to MACE or ACM associated with suboptimal statin use

Analysis	Statin use, *n* (%)		Time to MACE		Time to ACM	
	Suboptimal	Constant	HR (95% CI)	*P* value	HR (95% CI)	*P* value
Main analysis	156 (15.5)	849 (84.5)	2.10 (1.25–3.53)^1^	.005	2.46 (1.38–4.39)^2^	.003
Subgroup analyses						
Statin discontinuation/ nonadherence only	95 (10.1)	849 (89.9)	2.74 (1.49–5.04)^3^	.001	3.50 (1.69–7.23)^4^	.001
Statin dose reduction/ switch only	61 (6.7)	849 (93.3)	1.55 (0.75–3.20)^5^	.24	1.71 (0.72–4.04)^6^	.22
Main sensitivity analyses						
Including expanded nonadherence definition	272 (27.1)	733 (72.9)	1.75 (1.17–2.63)^7^	.007	1.75 (1.06–2.89)^8^	.030
Complete cases analysis	89 (12.3)	635 (87.7)	2.60 (1.58–4.28)^9^	<.001	3.41 (1.91–6.06)^10^	<.001

ACM, all-cause mortality; CI, confidence interval; MACE, major adverse cardiovascular events; HR, hazard ratio.

For each analysis (main, subgroup, and sensitivity analyses for both time to MACE and time to ACM), a multivariable covariate model was fitted before the suboptimal statin variable was added. Covariates with univariate *P* < .1 were entered into multivariable Cox proportional hazards modeling, with the final multivariable covariate model for each analysis chosen by forward stepwise (likelihood ratio) selection. All analyses selected to adjust for age ≥ 75 years, prior cardiovascular disease (previous myocardial infarction, stroke, transient ischemic attack, or peripheral artery disease), New York Heart Association functional class at Visit 2, and treatment with percutaneous coronary intervention or coronary artery bypass grafting surgery during baseline admission or within 30 days of discharge. Analysis 5 adjusted for no further covariates. Other covariates adjusted for in specific analyses were diabetes mellitus (analyses 1, 6, 7, 9, and 10); chronic kidney disease (analyses 2, 3, 4, and 8).
