# Supplementary material for: Investigating the prevalence, predictors, and prognosis of suboptimal statin use early after a non-ST elevation acute coronary syndrome
Source: J Clin Lipidol. 2017 Jan-Feb;11(1):204–14. doi: 10.1016/j.jacl.2016.12.007 (PMC5399750; doi:10.1016/j.jacl.2016.12.007)
Supplement: Supplementary Material [file mmc1.docx]

**Supplement for: Investigating the prevalence, predictors and prognosis of suboptimal statin use early after a non-ST elevation acute coronary syndrome**

**Supplement Methods**

This section provides additional information pertaining to:

- The prospective study
- Assessment of statin adherence
- Classification of suboptimal and constant statin users
- Covariate inclusion for this investigation
- Censoring
- Imputation of missing data during statistical analysis
- Sensitivity analyses

**Prospective study**

The investigation herein is based on clinical data from the Pharmacogenetics of Acute Coronary Syndrome (PhACS) prospective observational study, which ran from 2008-2013. The main inclusion criteria were hospitalisation with a non-ST elevation-ACS (both non-ST elevation myocardial infarction (NSTEMI) and unstable angina), and each potential participant had to be able to provide their own informed consent. NSTE-ACS was defined by either a positive troponin or appropriate electrocardiography (ECG) changes in the context of a history consistent with an ACS. Permissible ECG changes were: ST-depression, T-wave inversion, ST-segment flattening and transient self-resolving ST-elevation.

The exclusion criteria were:

- unwilling to participate
- inability to consent
- diagnosis of ST-elevation myocardial infarction (STEMI)
- a diagnosis or other pathology likely to account for symptoms or troponin rise
- a diagnosis or other pathology that may lead to death within one year other than cardiac (e.g. terminal lung cancer)
- no fixed address
- no current general practitioner
- not suitable, in the opinion of the Investigator, for participation in the study

The study continued until all patients had been followed up for at least 12 months. Patients were followed up at one and 12 months post index admission, representing visits two (V2) and three (V3), respectively. V2/V3 were conducted in person, or if not possible by telephone and/or a hospital case notes review (CNR). Subsequent visits were conducted annually thereafter by telephone and/or CNR. Patients that did not have a follow up visit during the last six months of study follow up had another CNR to ensure comprehensive clinical event capture. The general practitioners of patients missing drug data at V3 were contacted to collect V3 drug data and screen for cardiovascular events.

The endpoint clinical events were: death and non-fatal myocardial infarction (MI) or ischaemic stroke. Validation of non-fatal MIs was based on the algorithm used in TRITON-TIMI 38^1^, with the additional criterion that the treating physician team considered the raised cardiac biomarker (i.e. troponin) to be related to an ACS event, and therefore did not primarily diagnose and treat a non-ACS condition. Validation of non-fatal strokes was based on the HORIZONS-AMI definition, as an acute neurologic deficit lasting for over 24 hours, as classified by a physician, with supporting information, including brain images and neurologic/neurosurgical evaluation^2^. Non-fatal MIs and strokes were defined as those with no death within seven days and when death did occur within seven days and the death certificate was available, the MI/stroke was not listed within part I of the death certificate. Validation of cardiovascular deaths was based on the PLATO trial definition and included deaths due to cardiovascular disease (CVD), cerebrovascular deaths and any other deaths with no clearly documented non-cardiovascular cause^3^.

**Assessment of statin adherence**

Cardiac medication adherence was assessed using the Brief Medication Questionnaire (BMQ)^4^. The BMQ is an indirect method of determining medication adherence through patient self-reporting and incorporates three screens (eFigure. 1): a regimen, a belief and a recall screen. A small BMQ validation study (n=20) compared the BMQ to the Medication Events Monitoring System using ACEIs and determined that the regimen screen had a sensitivity and specificity of detecting repetitive non-adherence of 80% and 100%, respectively. However, it had 0% sensitivity for detecting sporadic non-adherence, giving an overall accuracy of 95%. The belief screen had sensitivities of detecting repetitive and sporadic non-adherence of 100% and 10%, respectively. However, it classed ~20% of adherent patients as repetitive non-adherent patients, giving an overall accuracy of 85%. The recall screen had sensitivities of detecting repetitive and sporadic non-adherence of 40% and 90%, respectively. However the respective specificities were only 40% and 80% ^4^.

For the main analysis, a pragmatic definition of statin non-adherence was used and taken to be present if a patient reported that they had missed one or more statin pills over the past week (from BMQ question 1e of the recall screen). For sensitivity analyses B and C4, the definition of non-adherence was expanded to include all of the available identifiers of potential non-adherence from the three screens^4^ (as listed in sensitivity analysis B).

**eFigure 1 The Brief Medication Questionnaire**

Please list below all of the cardiac medications you took in the past week. For each medication you list, please answer each of the questions in the box below.

**Question 1**

| **a. Medication name and strength (per tablet)** |  |
| --- | --- |
| **b. How many days did you take it?** |  |
| **c. How many times per day did you take it?** |  |
| **d. How many pills did you take each time?** |  |
| **e. How many times did you miss taking a pill?** |  |
| **f. For what reason were you taking it?** |  |
| **g. How well does the medicine work for you? 1=well; 2=okay; 3=not well** |  |

**Question 2** Do any of your medications bother you in any way?

Yes No *go to question 3*

| **How much did it bother you?** | | | | | **In what way did it bother you?** |
| --- | --- | --- | --- | --- | --- |
| **Medication name** | **A lot** | **Some** | **A little** | **Never** |  |

**Question 3** Below is a list of problems that people sometimes have with their medicine. Please check how hard it is for you to do each of the following:

|  | **Very hard** | **Somewhat hard** | **Not hard at all** | **Comment (which medicine)** |
| --- | --- | --- | --- | --- |
| **a. Open of close** the medication bottle |  |  |  |  |
| **b. Read the print** on the bottle |  |  |  |  |
| **c. Remember** to take all the pills |  |  |  |  |
| **d. Get your refills** in time |  |  |  |  |
| **e. Take so many pills** at the same time |  |  |  |  |

**Classification of Suboptimal and Constant Statin Users**

eTable 1 shows the estimated relative low density lipoprotein (LDL) cholesterol-lowering potencies of different statins across a range of clinically utilised doses. As the PhACS cohort is a secondary prevention cohort, the majority of patients were discharged on atorvastatin 80mg daily.

In this present study, constant statin users were defined as those who were discharged on a high potency statin, and remained on and adherent to this statin at V2. Atorvastatin 80mg, and rosuvastatin 20mg and 40mg were considered high potency statin therapy.

Suboptimal statin therapy was defined as occurring if a patient on high potency statin therapy at baseline discharged had discontinued, reduced the dose, switched to another statin of lower equivalent potency, and/or were non-adherent to statin therapy at V2. All doses at V2 with an estimated LDL-lowering potency less than atorvastatin 80mg/rosuvastatin 20mg daily were considered suboptimal.

**eTable 1 Statin doses estimated to lead to similar reductions in low-density lipoprotein cholesterol**

| **Fluvastatin** | **Lovastatin** | **Pravastatin** | **Simvastatin** | **Atorvastatin** | **Rosuvastatin** |
| --- | --- | --- | --- | --- | --- |
| 40 mg | 20 mg | 20 mg | 10 mg | - | - |
| 80 mg | 40 or 80 mg | 40 mg | 20 mg | 10 mg | - |
| - | 80 mg | 80 mg | 40 mg | 20 mg | 5 or 10 mg |
| - | - | - | 80 mg | 40 mg | - |
| - | - | - | - | 80 mg | 20 mg |
| - | - | - | - |  | 40 mg |

Based on Smith et al, 2006^5^

**Covariate inclusion**

The following were considered CYP3A4 inhibitors: amiodarone, clarithromycin, cyclosporine, diltiazem, erythromycin, itraconazole, protease inhibitors (indinavir, ritonavir, saquinavir), telithromycin and verapamil^6^. Levothyroxine and all CYP3A4 inhibitors, except diltiazem and verapamil, were not explicitly referred to in the case report form (CRF), but were accepted for this analysis if they had been noted in the CRF medication appendix during either baseline or V2. Their absence in the medication appendix was, for the purposes of this investigation, assumed to equate to not being prescribed/taken.

Patients were not explicitly questioned regarding muscular symptoms in PhACS. However, question 2 of the BMQ used at V2 asked if any medications bothered a patient, and if so, in what way; reports of bothersome muscular pains/cramps/aches/weakness whilst on statin therapy herein constituted muscular symptoms.

In this study, when investigating the risks of MACE and ACM associated with suboptimal statin use, an *a priori* decision was made to include all of the above covariates except for those covariates that had only been included because of their previous associations with statin-associated myotoxicity^6^. This is the reason that CYP3A4-inhibitors and levothyroxine, as well as actual muscular symptoms reported at V2, were excluded from MACE/ACM analyses.

**Censoring**

For the time to MACE analysis, participants were censored at the earliest of the date of non-CVD death or date of last recorded visit. For time to ACM, participants were censored at the date of the last recorded visit.

**Imputation of missing data during statistical analysis**

For the baseline demographic and comorbidity variables, there was <2% missing data. The percentage of missing cardiovascular and PPI drug data at V2 was 8.4-8.7%. The percentage of missing data for V2 statin status (high dose, low dose, discontinuation) was 9.4%, and ~19% of data were missing for each of V2 NYHA status, V2 muscular symptoms and adherence information. 9.6% of V2 dates were missing. Overall, 4.3% of data from included variables were known to be missing and 28.6% of cases had at least one missing value. The missing data was handled in three consecutive steps:

1. The median V2 follow up duration after baseline discharge, from all cases with a V2 date, was 30 days. Therefore for cases missing their V2 date (9.6% cases), it was calculated by adding 30 days onto the baseline discharge date of each case.
2. Manual imputation. For missing V2 covariate drug data, if the drug status at baseline and the next recorded visit (predominantly V3) remained unchanged (i.e. a patient remained on or off the drug) then this status was assumed for V2 and was manually imputed. For patients missing V2 statin status, if a patient remained on the same high dose statin therapy between baseline and the next recorded visit then this high dose statin status was manually imputed at V2. Where V2 statin adherence data was missing, if a patient remained on high dose statin therapy and was completely statin adherent at the next recorded follow up visit, then full adherence to high dose statin therapy at V2 was assumed. Only 1.2% of patients openly reported muscular symptoms at V2 and, given a missing data rate of ~19%, it was decided to assume that all patients missing this data did not have muscular symptoms. Following manual imputation, missing data rates for drug covariates, statin therapy, statin adherence and V2 muscular symptoms were reduced to 2.1-4.0, 4.9, 15.9% and 0.0%, respectively. Overall, 2.2% of data remained missing and 27% of cases still had at least one missing data value.
3. Multiple imputation. Following steps i and ii, the overall missing data rate was negligible, but as the BMQ had a high missing data rate, multiple imputation was undertaken, under the missing at random assumption. Although the missing data pattern had a monotonic trend, this pattern was incomplete and so all missing values were sampled using a fully conditional specification (FCS) method, which uses an iterative Markov chain Monte Carlo procedure (100 iterations), with generation of ten imputation datasets. Ordinal variables (statin status, statin adherence and NYHA classification) were treated as continuous variables and imputed using linear regression within the FCS procedure. Categorical variables were imputed by logistic regression. Covariate drug status at baseline and 12 months and NYHA status at 12 months were used as auxiliary variables to assist imputation.

**Sensitivity analyses**

To investigate the robustness of the results, several sensitivity analyses were undertaken.

**Sensitivity analyses A1-A2: Further assessments of the differences between the suboptimal and constant statin user groups:**

In sensitivity analysis A1, a complete cases subcohort was used to describe the univariate (sensitivity analysis A1.1, eTable 2) and multivariable (A1.2, logistic regression, eTable 3) differences in covariate prevalence between the suboptimal and constant statin user groups. The complete cases cohort excluded all patients with any missing data that required imputation by any of the implemented methods. In the main imputed dataset, sensitivity analysis A2 (eTable 4) investigated the risk of suboptimal statin use with muscular symptoms if this variable also underwent multiple imputation.

**Sensitivity analyses B-F: Further assessments of the risks of MACE and ACM associated with suboptimal statin therapy**

**Sensitivity analysis B**

Sensitivity analysis B referred to the expanded full statin non-adherence definition, incorporating: patients that missed at least one statin pill (BMQ Qu. 1e), took a statin for six or less days (Qu. 1b) (both from regimen screen), reported that the statin did not work well for them or they did not know (Qu. 1g), found that the statin bothered them at least a little (Qu. 2) (both from belief screen) and those that found it at least somewhat hard to remember to take all of their pills (Qu. 3c from the recall screen), respectively (eTable 5).

**Sensitivity analyses C1-C4**

Within the complete cases cohort (all patients with any missing data excluded), sensitivity analysis C1 assessed the risks of time to MACE and ACM for all patients with suboptimal statin therapy, C2 assessed just those who had discontinued or were statin non-adherent, C3 assessed just those who had reduced the dose or switched statin (but were adherent), and C4 assessed the expanded statin non-adherence definition, compared to constant statin users (eTable 5).

**Sensitivity analyses D1 and D2**

For those variables that conspicuously disobeyed the proportional hazards assumption for the full follow up duration upon inspection of unadjusted Kaplan-Meier curves (V2 P2Y_12_ inhibitor use for MACE, and sex for ACM), their impact on MACE and ACM was first investigated via univariate and multivariable logistic regression analyses (utilising all endpoint events), as has been previously recommended^7^ (D1, eTable 6). Secondly, they were investigated by limiting follow up to 11 months after V2 (essentially 12 months after baseline discharge), because the proportional hazards assumption was upheld for these variables within this shorter duration (D2, eTable 7).

**Sensitivity analyses E1 and E2**

To ensure that the variables that differed significantly between the suboptimal and constant statin user groups at visit 2 did not affect the adjusted associations between statin utilisation and times to MACE or ACM, multivariable models including these variables were performed. As two of these variables (patient sex and P2Y_12_ inhibitor status at visit 2) did not meet the proportional hazards assumption, both multivariable logistic regression (eTable 8) and Cox proportional hazards modelling (eTable 9) censored at 11 months after visit 2 were carried out.

**Sensitivity analyses F1 and F2**

It is possible that statin prescription alterations early after discharge simply identify patients that received more medical attention because they are more ill. Thus, to investigate healthy user bias within this study’s cohort, PPI prescription changes were considered. As many patients were not discharged from their index NSTE-ACS admission on a PPI, constant PPI status was defined as: not on a PPI or on the same PPI and dose at both baseline and V2. PPI changers were defined as: patients that started, stopped or switched PPI or increased or decreased their PPI dose. When comparing constant to PPI changers for the full follow up duration, the proportional hazards assumption was not met and therefore, healthy user bias was assessed as above using both logistic regression (E1, eTable 10) and limiting follow up to 11 months after V2 (E2, eTable 11).

Supplement Results

**eTable 2 Characteristics of suboptimal and constant statin user groups using only participants with complete data (Sensitivity Analysis A1.1).**

| **Variable** | **Suboptimal Statin therapy** | **Constant Statin users** | **Univariate p-value** |
| --- | --- | --- | --- |
| **Patients (%)** | 89 (12.3) | 635 (87.7) |  |
| **Demographics** | | | |
| Age ≥ 75, n (%) | 19 (21.3) | 141 (18.0) | 0.44 |
| Men, n (%) | 59 (66.3) | 439 (77.6) | **0.019** |
| BMI ≥ 30, n (%) | 32 (36.0) | 217 (34.2) | 0.74 |
| **Medical History, n (%)** | | | |
| Hypertension | 55 (61.8) | 361 (56.9) | 0.38 |
| Hyperlipidaemia | 45 (50.6) | 347 (54.6) | 0.47 |
| Diabetes mellitus | 25 (28.1) | 129 (20.3) | 0.093 |
| Ever smoked | 68 (76.4) | 442 (69.6) | 0.19 |
| CKD (Cr>150µmol/L) | 8 (9.0) | 36 (5.7) | 0.22 |
| COPD | 8 (9.0) | 52 (8.2) | 0.78 |
| Prior CVD^1^ | 29 (32.6) | 221 (34.8) | 0.68 |
| On Statin prior to index admission | 43 (48.3) | 286 (45.0) | 0.56 |
| **Diagnosis, n (%)** | | | |
| Troponin-raised NSTE-ACS^2^ | 86 (96.6) | 620 (97.6) | 0.48 |
| Normal troponin NSTE-ACS | 3 (3.4) | 15 (2.4) | - |
| **Treatment, n (%)** | | | |
| PCI/CABG | 43 (48.3) | 302 (47.6) | 0.89 |
| Discharged on Atorvastatin 80mg daily^2^ | 89 (100.0) | 630 (99.2) | >0.99 |
| **NYHA Functional Classification at Visit 2, n (%)^2^** | | | |
| Class I | 48 (53.9) | 343 (54.0) | 0.75 |
| Class II | 32 (36.0) | 235 (37.0) |  |
| Class III | 9 (10.1) | 49 (7.7) |  |
| Class IV | 0 (0.0) | 8 (1.3) |  |
| **Drugs at Visit 2, n (%)** | | | |
| Aspirin | 82 (92.1) | 595 (93.7) | 0.57 |
| P2Y_12_ inhibitor | 71 (79.8) | 549 (86.5) | 0.092 |
| Beta blocker | 67 (75.3) | 553 (87.1) | **0.003** |
| ACEI/ARB | 70 (78.7) | 536 (84.4) | 0.17 |
| Warfarin^2^ | 3 (3.4) | 33 (5.2) | 0.61 |
| Proton pump inhibitor | 39 (43.8) | 265 (41.7) | 0.71 |
| CYP3A4-inhibitors | 13 (14.6) | 44 (6.9) | **0.012** |
| Levothyroxine^2^ | 3 (3.4) | 29 (4.6) | 0.79 |
| Muscular symptoms at V2(%)^2^ | 4 (4.5) | 7 (1.1) | **0.036** |

1= Prior CVD encompasses past myocardial infarction, stroke, transient ischaemic attack or peripheral artery disease; 2 = Fisher’s Exact test used. Unless otherwise stated, Pearson chi square statistical testing was used here in the single dataset complete cases subcohort

**eTable 3 Factors associated with an increased risk of suboptimal statin occurrence in logistic regression using only participants with complete data (Sensitivity Analysis A1.2).**

| **Risk factor** | **Multivariable adjusted analysis^1^** | |
| --- | --- | --- |
|  | **OR (95% CI)** | **p-value** |
| Muscular symptoms | 4.89 (1.38-17.28) | 0.014 |
| Sex (F vs M) | 1.66 (1.02-2.70) | 0.040 |
| Beta blocker at V2 | 0.45 (0.26-0.78) | 0.004 |

^1^ = Variables with p<0.1 in univariate analysis (muscular symptoms, CYP3A4-inhibitors, diabetes mellitus, sex, P2Y_12_ inhibitor use and beta blocker use at Visit 2) were entered into multivariable logistic regression modelling using the forwards likelihood ratio method to select the final presented model.

**eTable 4 Factors associated with an increased risk of suboptimal statin use if muscular symptoms also had multiple imputation in the main dataset (Sensitivity Analysis A2).**

| **Risk factor** | **Suboptimal Statin therapy n (%)** | **Constant Statin users n (%)** | **Multivariable adjusted analysis^1^** | |
| --- | --- | --- | --- | --- |
|  |  |  | **OR (95% CI)** | **p-value** |
| Muscular symptoms^2^ | 25 (16.0) | 47 (5.5) | 3.43 (1.28-9.20) | 0.017 |
| Sex (F vs M) | M: 102 (65.4) | M: 660 (77.4) | 1.74 (1.13-2.69) | 0.012 |
| P2Y_12_ inhibitor at V2 | 122 (78.2) | 738 (86.9) | 0.54 (0.34-0.87) | 0.011 |
| Beta blocker at V2 | 119 (76.3) | 725 (85.4) | 0.60 (0.36-1.00) | 0.050 |

^1^ = Variables with p<0.1 in univariate analysis using imputed data (muscular symptoms, CYP3A4-inhibitors, diabetes mellitus, sex, P2Y_12_ inhibitor use and beta blocker use at Visit 2) were entered into multivariable logistic regression modelling using the forwards likelihood ratio method to select the final presented model.

^2^ = It was evident that the multiple imputation for muscular symptoms increased the apparent prevalence of symptoms, and therefore multiple imputation was not used for this covariate in the main analysis

eTable 5 Summary table of all analyses of the multivariable adjusted risks of MACE, or ACM, associated with suboptimal statin users compared to constant statin users

| **Analysis** | **Description** | | **MACE** | | **ACM** | |
| --- | --- | --- | --- | --- | --- | --- |
|  |  |  | **Risk Estimate (95% CI)** | **p-value** | **Risk Estimate (95% CI)** | **p-value** |
| Main Analysis | Suboptimal statin users (n=156) | | HR 2.10 (1.25-3.53)^1^ | 0.005 | HR 2.46 (1.38-4.39)^2^ | 0.003 |
| Statin discontinuation/  non-adherence (n=95) | | | HR 2.74 (1.49-5.04)^3^ | 0.001 | HR 3.50 (1.69-7.23)^4^ | 0.001 |
| Statin dose reduction/  Switch (n=61) | | | HR 1.55 (0.75-3.20)^5^ | 0.24 | HR 1.71 (0.72-4.04)^6^ | 0.22 |
| Sen analysis B | | Expanded statin non-adherence definition (n=272) | HR 1.75 (1.17-2.63)^7^ | 0.007 | HR 1.75 (1.06-2.89)^8^ | 0.030 |
| Sen analysis C: Participants with complete data only. Constant statin users, n=635, versus: | | | | | | |
| C1 | All suboptimal statin cases (n=89) | | HR 2.60 (1.58-4.28)^9^ | <0.001 | HR 3.41 (1.91-6.06)^10^ | <0.001 |
| C2 | Statin discontinuation/  non-adherence (n=51) | | HR 3.94 (2.07-7.48)^11^ | <0.001 | HR 5.06 (2.35-10.90)^12^ | <0.001 |
| C3 | Statin dose reduction/  switch (n=38) | | HR 1.84 (0.91-3.71)^13^ | 0.090 | HR 2.41 (1.07-5.40)^14^ | 0.034 |
| C4 | Expanded statin non-adherence definition (n=178) | | HR 1.83 (1.19-2.84)^15^ | 0.006 | HR 1.85 (1.09-3.12)^16^ | 0.022 |
| Sen analyses D, E and F: suboptimal statin users, n=156, versus constant statin users, n=849 | | | | | | |
| Sen analysis D: Covariates that did not meet the proportional hazards assumption during full follow up included: | | | | | | |
| D1 | Logistic regression | | OR 2.61 (1.50-4.54)^17^ | 0.001 | OR 3.18 (1.68-6.01)^18^ | <0.001 |
| D2 | Censor at 11 months | | HR 3.54 (1.90-6.61)^19^ | <0.001 | HR 5.11 (2.32-11.25)^20^ | <0.001 |
| Sen analysis E: variables that differed significantly between suboptimal and constant statin users at visit 2 included: | | | | | | |
| E1 | Logistic regression | | OR 2.65 (1.50-4.65)^21^ | 0.001 | OR 3.17 (1.67-6.04)^22^ | <0.001 |
| E2 | Censor at 11 months | | HR 3.49 (1.85-6.58)^23^ | <0.001 | HR 5.07 (2.26-11.40)^24^ | <0.001 |
| Sen analysis E: Healthy user assessment variable included: | | | | | | |
| F1 | logistic regression | | OR 2.53 (1.46-4.37)^25^ | 0.001 | OR 3.39 (1.80-6.39)^26^ | <0.001 |
| F2 | Censor at 11 months | | HR 3.55 (1.91-6.60)^27^ | <0.001 | HR 5.52 (2.56-11.89)^28^ | <0.001 |

HR = hazard ratio; OR = odds ratio.

For each analysis, a multivariable covariate model was fitted before the suboptimal statin variable and any additional pre-specified variables (e.g. healthy user assessment variable in analyses F1 and F2) were added. Covariates with univariate p<0.1 were entered into multivariable modelling, with the final multivariable covariate model for each analysis chosen by forwards stepwise (likelihood ratio) selection. All analyses adjusted for age ≥ 75 and prior cardiovascular disease (previous myocardial infarction, stroke, transient ischaemic attack or peripheral artery disease). All analyses adjusted for treatment with percutaneous coronary intervention or coronary artery bypass grafting surgery during baseline admission or within 30 days of discharge except analyses 20, 24 and 28. All analyses adjusted for New York Heart Association functional class at Visit 2 except analysis 12. Other covariates adjusted for in specific analyses were: diabetes mellitus (analyses 1, 6, 7, 9-16, 19, 23, 27); chronic kidney disease (analyses 2, 3, 4, 8, 12, 17, 18, 21, 22, 25, 26); P2Y_12_ inhibitor at V2 (analyses 17, 19, 21-24); sex (analyses 18, 20, 21-24); aspirin at V2 (analyses 18, 22, 26); beta blocker at V2 (analyses 21-24); muscular symptoms (analyses 21-24); healthy user assessment (analyses 25-28).

### It was important to investigate further the variables that did not obey the Cox proportional hazards assumption for full study follow up (V2 P2Y_12_ inhibitor use for MACE, and sex for ACM) to determine their impact, if any, on the main study results. The majority of known P2Y_12_ inhibitor prescriptions were for clopidogrel (>98%). In both adjusted logistic regression (sensitivity analysis D1) and when follow up was censored at 11 months post V2 (sensitivity analysis D2), suboptimal statin use remained significantly associated with both MACE and ACM, and importantly neither of these two covariates were significantly associated with either endpoint (eTables 6, 7).

eTable 6 Risk of MACE and ACM identified in logistic regression analysis including variables that did not meet the proportional hazards assumption during all study follow up (Sensitivity Analysis D1)

| **Variable** | **MACE** | | **ACM** | |
| --- | --- | --- | --- | --- |
|  | **OR (95% CI)** | **p-value** | **OR (95% CI)** | **p-value** |
| **Univariate analysis:** | | | | |
| P2Y_12_ inhibitor at V2^1^ | 1.01 (0.57-1.79) | 0.97 | - | - |
| Sex (F vs M)^1^ | - | - | 1.76 (1.07-2.87) | 0.025 |
| **Multivariable adjusted analysis^2^:** | | | | |
| **Suboptimal Statin therapy** | **2.61 (1.50-4.54)** | **0.001** | **3.18 (1.68-6.01)** | **<0.001** |
| Age ≥ 75 | 2.49 (1.57-3.93) | <0.001 | 3.61 (2.09-6.22) | <0.001 |
| NYHA | 1.81 (1.34-2.44) | <0.001 | 1.91 (1.30-2.79) | 0.001 |
| Prior CVD | 2.52 (1.61-3.93) | <0.001 | 2.93 (1.67-5.17) | <0.001 |
| Treatment with PCI/CABG | 0.57 (0.36-0.91) | 0.018 | 0.47 (0.26-0.87) | 0.016 |
| Chronic kidney disease | 1.99 (1.04-3.82) | 0.039 | 2.88 (1.41-5.87) | 0.004 |
| Aspirin at V2 | - | - | 0.40 (0.20-0.80) | 0.010 |
| **P2Y_12_ inhibitor at V2^1^** | **1.51 (0.80-2.85)** | **0.21** | - | - |
| **Sex (F vs M)^1^** | - | - | **1.42 (0.81-2.51)** | **0.22** |

^1^ = Covariates that did not meet the proportional hazards assumption during the full follow up duration for time to major adverse cardiovascular events (visit 2 P2Y_12_ status) or time to all-cause mortality (sex).

^2^ = For the multivariable logistic regression MACE/ACM analyses, covariates with univariate p<0.1 were entered into multivariable logistic regression modelling using the forwards likelihood ratio method to select the covariate model (variables not in bold font). After these MACE and ACM logistic regression covariate models were selected, the variables in bold were entered into the relevant model to produce the presented results.

### eTable 7 Risk of time to MACE and ACM identified using survival analysis with follow up censored at 11 months after Visit 2 and including variables that did not meet the proportional hazards assumption during all study follow up (Sensitivity Analysis D2)

| **Variable** | **MACE** | | **ACM** | |
| --- | --- | --- | --- | --- |
|  | **HR (95% CI)** | **p-value** | **HR (95% CI)** | **p-value** |
| **Univariate analysis:** | | | | |
| P2Y_12_ inhibitor at V2^1^ | 0.82 (0.41-1.61) | 0.56 | - | - |
| Sex (F vs M)^1^ | - | - | 1.61 (0.78-3.32) | 0.20 |
| **Multivariable adjusted analysis^2^:** | | | | |
| **Suboptimal Statin therapy** | **3.54 (1.90-6.61)** | **<0.001** | **5.11 (2.32-11.25)** | **<0.001** |
| Age ≥ 75 | 2.11 (1.22-3.67) | 0.008 | 3.15 (1.51-6.55) | 0.002 |
| NYHA | 1.47 (0.999-2.16) | 0.050 | 2.12 (1.27-3.54) | 0.004 |
| Treatment with PCI/CABG | 0.55 (0.31-0.99) | 0.049 | - | - |
| Diabetes mellitus | 1.76 (1.01-3.07) | 0.046 | - | - |
| Prior CVD | 2.17 (1.22-3.86) | 0.008 | 2.59 (1.21-5.57) | 0.015 |
| **P2Y_12_ inhibitor at V2^1^** | **1.27 (0.62-2.61)** | **0.52** | - | - |
| **Sex (F vs M)^1^** | - | - | **1.21 (0.58-2.55)** | **0.61** |

^1^ = Covariates that did not meet the proportional hazards assumption during the full follow up duration for time to major adverse cardiovascular events (visit 2 P2Y_12_ status) or time to all-cause mortality (sex), but they did both meet the assumption when censored at 11 months post Visit 2.

^2^ = For the multivariable Cox regression time to MACE/ACM analyses, covariates with p<0.1 in univariate Cox analysis were entered into multivariable Cox regression modelling (follow up to 11 months after Visit 2) using the forwards likelihood ratio method to select the covariate model (variables not in bold font). After these time to MACE and ACM covariate models were selected, the variables in bold were entered into the relevant model to produce the presented results.

As reported in eTables 8 and 9, the variables that differed significantly between suboptimal and constant statin users at visit 2 had no impact on the association of suboptimal statin therapy with MACE or ACM. Furthermore, none of the four variables (muscular symptoms, sex, beta blocker or P2Y_12_ inhibitor therapy at visit 2) were associated themselves with either endpoint. Please note, although muscular symptoms were not in the list of variables pre-specified to be used for investigating associations with MACE and ACM (as stipulated in the Material and methods covariates section), it was decided to include it in this sensitivity analysis because muscular symptoms differed between suboptimal and constant statin users.

eTable 8 Risk of MACE and ACM identified in multivariable logistic regression analysis including variables that differed significantly between suboptimal and constant statin users at Visit 2 (Sensitivity Analysis E1)

| **Variable** | **MACE** | | **ACM** | |
| --- | --- | --- | --- | --- |
|  | **OR (95% CI)** | **p-value** | **OR (95% CI)** | **p-value** |
| **Suboptimal Statin therapy** | **2.65 (1.50-4.65)** | **0.001** | **3.17 (1.67-6.04)** | **<0.001** |
| Age ≥ 75 | 2.47 (1.55-3.91) | <0.001 | 3.61 (2.08-6.25) | <0.001 |
| NYHA | 1.81 (1.34-2.45) | <0.001 | 1.91 (1.30-2.79) | 0.001 |
| Prior CVD | 2.51 (1.60-3.92) | <0.001 | 2.94 (1.67-5.19) | <0.001 |
| Treatment with PCI/CABG | 0.57 (0.36-0.90) | 0.016 | 0.47 (0.26-0.87) | 0.017 |
| Chronic kidney disease | 1.96 (1.02-3.78) | 0.044 | 2.91 (1.42-5.95) | 0.003 |
| Aspirin at V2 | - | - | 0.40 (0.19-0.82) | 0.013 |
| **Muscular symptoms** | **0.00 (0.00-0.00)** | **>0.99** | **1.25 (0.14-11.40)** | **0.84** |
| **Sex (F vs M)** | **1.10 (0.68-1.78)** | **0.69** | **1.43 (0.81-2.53)** | **0.22** |
| **Beta Blocker inhibitor at V2** | **1.08 (0.61-1.92)** | **0.79** | **1.05 (0.53-2.10)** | **0.89** |
| **P2Y_12_ inhibitor at V2** | **1.50 (0.79-2.85)** | **0.21** | **0.96 (0.48-1.93)** | **0.91** |

For the multivariable logistic regression MACE/ACM analyses, covariates with univariate p<0.1 were entered into multivariable logistic regression modelling using the forwards likelihood ratio method to select the covariate model (variables not in bold font). After these MACE and ACM logistic regression covariate models were selected, the variables in bold were entered into the relevant model to produce the presented results.

### eTable 9 Risk of time to MACE and ACM identified using multivariable survival analysis with follow up censored at 11 months after Visit 2, and including variables that differed significantly between suboptimal and constant statin users at Visit 2 (Sensitivity Analysis E2)

| **Variable** | **MACE** | | **ACM** | |
| --- | --- | --- | --- | --- |
|  | **HR (95% CI)** | **p-value** | **HR (95% CI)** | **p-value** |
| **Suboptimal Statin therapy** | **3.49 (1.85-6.58)** | **<0.001** | **5.07 (2.26-11.40)** | **<0.001** |
| Age ≥ 75 | 2.09 (1.20-3.66) | 0.010 | 2.88 (1.35-6.15) | 0.006 |
| NYHA | 1.46 (0.99-2.15) | 0.057 | 2.09 (1.25-3.49) | 0.006 |
| Treatment with PCI/CABG | 0.56 (0.31-1.01) | 0.054 | - | - |
| Diabetes mellitus | 1.79 (1.02-3.14) | 0.041 | - | - |
| Prior CVD | 2.16 (1.22-3.84) | 0.009 | 2.63 (1.23-5.62) | 0.013 |
| **Muscular symptoms** | **0.00 (0.00-0.00)** | **>0.99** | **0.00 (0.00-0.00)** | **>0.99** |
| **Sex (F vs M)** | **1.26 (0.71-2.23)** | **0.43** | **1.26 (0.59-2.65)** | **0.55** |
| **Beta Blocker inhibitor at V2** | **1.00 (0.50-2.01)** | **>0.99** | **1.01 (0.41-2.51)** | **0.98** |
| **P2Y_12_ inhibitor at V2** | **1.26 (0.61-2.62)** | **0.53** | **0.62 (0.27-1.40)** | **0.25** |

For the multivariable Cox regression time to MACE/ACM analyses, covariates with p<0.1 in univariate Cox analysis were entered into multivariable Cox regression modelling (follow up to 11 months after Visit 2) using the forwards likelihood ratio method to select the covariate model (variables not in bold font). After these time to MACE and ACM covariate models were selected, the variables in bold were entered into the relevant model to produce the presented results.

When the healthy user variable was included (sensitivity analyses F1, F2), suboptimal statin use remained a statistically significant predictor of MACE and ACM. The healthy user variable was not associated with MACE. Although the healthy user variable was nominally associated with time to ACM up to 11 months after Visit 2 (eTable 11), it was not significant after correction for multiple testing (p-value threshold 0.025). Furthermore, it was not significantly associated with ACM risk in multivariable logistic regression analysis which includes all recorded endpoints (eTable 10).

eTable 10 Assessment of potential healthy user bias on the risk of MACE or ACM associated with suboptimal statin use using logistic regression analysis (Sensitivity Analysis F1)

| **Variable** | **MACE** | | **ACM** | |
| --- | --- | --- | --- | --- |
|  | **OR (95% CI)** | **p-value** | **OR (95% CI)** | **p-value** |
| **Univariate analysis:** | | | | |
| Healthy User Assessment^1^ | 1.08 (0.66-1.78) | 0.76 | 1.60 (0.94-2.73) | 0.083 |
| **Multivariable adjusted analysis^2^:** | | | | |
| **Suboptimal Statin therapy** | **2.53 (1.46-4.37)** | **0.001** | **3.39 (1.80-6.39)** | **<0.001** |
| **Healthy User Assessment^1^** | **1.01 (0.58-1.74)** | **0.99** | **1.59 (0.85-2.98)** | **0.15** |
| Age ≥ 75 | 2.38 (1.51-3.76) | <0.001 | 3.62 (2.10-6.26) | <0.001 |
| NYHA | 1.78 (1.32-2.39) | <0.001 | 1.89 (1.29-2.78) | 0.001 |
| Treatment with PCI/CABG | 0.57 (0.36-0.91) | 0.018 | 0.45 (0.24-0.83) | 0.011 |
| Prior CVD | 2.55 (1.63-3.97) | <0.001 | 2.97 (1.68-5.24) | <0.001 |
| Chronic kidney disease | 2.00 (1.05-3.83) | 0.036 | 2.92 (1.44-5.95) | 0.003 |
| Aspirin at V2 | - | - | 0.41 (0.20-0.83) | 0.014 |

^1^ = Healthy user assessment is a variable comparing patients with constant proton pump inhibitor (PPI) use, defined as not on a PPI or on the same PPI and dose at both baseline and V2, compared to PPI changers, defined as patients that started, stopped or switched PPI or increased or decreased their PPI dose.

^2^ = For the multivariable logistic regression MACE/ACM analyses, covariates with univariate p<0.1 were entered into multivariable logistic regression modelling using the forwards likelihood ratio method to select the covariate model (variables not in bold font). After these MACE and ACM logistic regression covariate models were selected, the variables in bold were entered into each model to produce the presented results.

eTable 11 Assessment of potential healthy user bias on the risks of time to MACE or ACM associated with suboptimal statin with follow up censored at 11 months after Visit 2 (Sensitivity Analysis F2)

| **Variable** | **Time to MACE** | | **Time to ACM** | |
| --- | --- | --- | --- | --- |
|  | **HR (95% CI)** | **p-value** | **HR (95% CI)** | **p-value** |
| **Univariate analysis:** | | | | |
| Healthy User Assessment^1^ | 1.39 (0.76-2.54) | 0.28 | 2.25 (1.09-4.65) | 0.029 |
| **Multivariable adjusted analysis^2^:** | | | | |
| **Healthy User Assessment^1^** | **1.42 (0.77-2.61)** | **0.27** | **2.24 (1.05-4.77)** | **0.037** |
| **Suboptimal Statin therapy** | **3.55 (1.91-6.60)** | **<0.001** | **5.52 (2.56-11.89)** | **<0.001** |
| Age ≥ 75 | 2.03 (1.17-3.50) | 0.011 | 3.02 (1.46-6.27) | 0.003 |
| NYHA | 1.46 (0.99-2.14) | 0.054 | 2.09 (1.28-3.43) | 0.004 |
| Treatment with PCI/CABG | 0.55 (0.31-0.99) | 0.048 | - | - |
| Prior CVD | 2.23 (1.26-3.95) | 0.006 | 2.78 (1.29-6.01) | 0.009 |
| Diabetes mellitus | 1.73 (0.997-2.99) | 0.051 | - | - |

^1^ = Healthy user assessment is a variable comparing patients with constant proton pump inhibitor (PPI) use, defined as not on a PPI or on the same PPI and dose at both baseline and V2, compared to PPI changers, defined as patients that started, stopped or switched PPI or increased or decreased their PPI dose.

^2^ = For the multivariable Cox regression time to MACE/ACM analyses, covariates with p<0.1 in univariate Cox analysis were entered into multivariable Cox regression modelling (follow up to 11 months after Visit 2) using the forwards likelihood ratio method to select the covariate model (variables not in bold font). After these time to MACE and ACM covariate models were selected, the variables in bold were entered into both models to produce the presented results.

**Supplement References**

1 Wiviott SD, Antman EM, Gibson CM, et al. Evaluation of prasugrel compared with clopidogrel in patients with acute coronary syndromes: design and rationale for the TRial to assess Improvement in Therapeutic Outcomes by optimizing platelet InhibitioN with prasugrel Thrombolysis In Myocardial Infarction 38 (TRITON-TIMI 38). Am Heart J. 2006 Oct;152(4):627-35.

2 Mehran R, Brodie B, Cox DA, et al. The Harmonizing Outcomes with RevasculariZatiON and Stents in Acute Myocardial Infarction (HORIZONS-AMI) Trial: study design and rationale. Am Heart J. 2008 Jul;156(1):44-56.

3 James S, Akerblom A, Cannon CP, et al. Comparison of ticagrelor, the first reversible oral P2Y(12) receptor antagonist, with clopidogrel in patients with acute coronary syndromes: Rationale, design, and baseline characteristics of the PLATelet inhibition and patient Outcomes (PLATO) trial. Am Heart J. 2009 Apr;157(4):599-605.

4 Svarstad BL, Chewning BA, Sleath BL, Claesson C. The Brief Medication Questionnaire: a tool for screening patient adherence and barriers to adherence. Patient Educ Couns. 1999 Jun;37(2):113-24.

5 Smith MEB, Lee NJ, Haney E, Carson S. Drug Class Review: HMG-CoA Reductase Inhibitors (Statins) and Fixed-dose Combination Products Containing a Statin: Final Report Update 5. 2009 [cited; Available from: <http://www.ncbi.nlm.nih.gov/books/NBK47273/pdf/Bookshelf_NBK47273.pdf>

6 Alfirevic A, Neely D, Armitage J, et al. Phenotype standardization for statin-induced myotoxicity. Clin Pharmacol Ther. 2014 Oct;96(4):470-6.

7 Wachtel MS, Yang S. Odds of death after glioblastoma diagnosis in the United States by chemotherapeutic era. Cancer Med. 2014 Jun;3(3):660-6.
